# Response to letter to the editor

**DOI:** 10.1002/clc.23872

**Published:** 2022-06-14

**Authors:** Aadhavi Sridharan, Ayan R. Patel

**Affiliations:** ^1^ Cardiovascular Imaging and Hemodynamic Laboratory Tufts Medical Center Boston Massachusetts USA; ^2^ Heart Failure and Cardiac Transplant Program Tufts Medical Center Boston Massachusetts USA

We welcome and appreciate the comments raised by Ghodsi et al. related to our recent publication “Accuracy of echocardiographic estimations of right heart pressures in adult heart transplant recipients.”^1^ In this article, we analyzed the correlation between noninvasive echocardiographic estimation of right atrial pressure (RAP) and pulmonary artery systolic pressures (PASP) and those derived from invasive right heart catheterization in adult heart transplant recipients. Our findings demonstrated no significant correlation between echocardiographic estimation and invasively measured RAP and only a modest correlation between echocardiographic estimation and invasively measured PASP in heart transplant recipients.

In response to the comments raised in this letter, we present a few clarifications here. First, all heart transplant patients included in the study underwent bicaval and left atrial anastomoses with complete removal of the recipient's right atrium. Therefore, while there is complete preservation of the donor's right atrium with this surgical technique, as we postulate in the discussion, whether such mechanical disruption of the inferior vena cava (IVC) with caval anastomosis interferes with RAP estimations remains to be studied.

The primary objective of our analysis was to evaluate the accuracy of echocardiographic measurements of IVC size and collapsibility as well as tricuspid regurgitation gradient in the assessment of right heart filling pressures in this special patient population, as per current American Society of Echocardiography (ASE) guidelines.^2^ Our main finding of poor to modest correlation between echocardiographic estimates and invasively measured hemodynamics thus supports further refinement of these guidelines for accurate echocardiographic estimation of right heart pressures in heart transplant recipients. While there certainly may be other echocardiographic measures (such as tricuspid *E*/*e*ʹ ratio and hepatic vein flow), as the authors suggest, that may better correlate with invasive measurements, the purpose of our investigation was focused on examining the performance of IVC parameters for estimation of right heart pressures in this population. Similarly, assessing and adjusting for clinical biomarkers such as brain natriuretic peptide was beyond the scope of our current study. Finally, cardiac magnetic resonance imaging (MRI) is a complementary noninvasive modality that can be used to image atrial structure, however its use is currently limited due to cost, availability, prolonged imaging times and patient factors such as contraindications to MRI.

The findings of our study suggest that current ASE guidelines for noninvasive estimations of right heart pressures may not be reliable in heart transplant recipients; this is clinically important because right heart pressures have prognostic value and timely recognition of elevated right heart pressure is critical to prevent unfavorable outcomes in this patient population. We positively agree with the overall observations presented in the letter that alternative echocardiographic measures and/or alternative imaging techniques for noninvasive estimation of right heart pressures need to be investigated and validated in this unique patient population.
